# Evaluation of the Effect of Electronic Cigarette Devices/Vape on the Color of Dental Ceramics: An In Vitro Investigation

**DOI:** 10.3390/ma16113977

**Published:** 2023-05-26

**Authors:** Ghada Alrabeah, Syed Rashid Habib, Nawaf M. Alamro, Meshari A. Alzaaqi

**Affiliations:** 1 Department of Prosthetic Dental Sciences, College of Dentistry, King Saud University, P.O. Box 60169, Riyadh 11545, Saudi Arabia; 2 Intern, College of Dentistry, King Saud University, P.O. Box 60169, Riyadh 11545, Saudi Arabiaal-zaaqi@hotmail.com (M.A.A.)

**Keywords:** e-cigarettes, vape, vaping, dental shade, dental ceramics, spectrophotometer, tooth shade

## Abstract

The use of vaping or electronic cigarette devices (ECDs) has recently increased as an alternative to conventional tobacco smoking products. By recording the CIELAB coordinates (L*a*b*) and computing the total color difference (ΔE) values using a spectrophotometer, the effect of ECDs on contemporary aesthetic dental ceramics was investigated in this in-vitro study. A total of seventy-five (N = 75) specimens from five different (*n* = 15) dental ceramic materials (Pressable ceramics (PEmax); Pressed and layered ceramics (LEmax); Layered zirconia (LZr); Monolithic zirconia (MZr) and Porcelain fused to metal (PFM)) were prepared and exposed to aerosols produced by the ECDs. The color assessment was performed using a spectrophotometer at six time intervals (0 = baseline; 250-puff exposures; 500-puff exposures; 750-puff exposures; 1000-puff exposures; 1250-puff exposures; and 1500-puff exposures). By recording L*a*b* and computing total color difference (ΔE) values, the data were processed. A one-way ANOVA and Tukey procedure for pairwise comparisons were used to assess color differences between tested ceramics (*p* < 0.05). All test materials demonstrated significant color differences (ΔE) after exposure to vaping (*p* < 0.05). The LZr group displayed noticeably high ΔE values at all the distinct puff exposure intervals, with the highest ΔE value of (13.67) after 1500 puffs. The lowest (ΔE) values were observed in the PFM group after 250 and 500 puffs (0.85 and 0.97, respectively). With the exception of the group PEmax (*p* = 0.999), all groups produced readings of “ΔE” that indicated significant differences (*p* < 0.05) at various degrees of puff exposures. ECDs can noticeably alter the color of the dental ceramics affecting the esthetics of the patients. All the materials tested demonstrated significant color changes (ΔE > 3.33) above the clinically acceptable threshold, except for the PFM and PEmax group (ΔE < 3.33) which showed color stability after exposure to the ECDs.

## 1. Introduction

Esthetic dentistry has become an integral part of the total cosmetic treatment for facial beauty. Patients’ high demand for cosmetic restorations has encouraged manufacturers to continuously produce new restorative materials with advanced esthetic properties that satisfy patients’ desires and meet their expectations [1]. Dental ceramics are among the highly esthetic treatment modalities for anterior and posterior teeth restorations [2]. Such tooth-colored materials possess distinguished esthetic and mechanical properties that enable them to withstand the dynamic oral environment [2,3]. Within this hostile environment, teeth and existing restorations are continuously exposed to various stimuli that could influence their appearance [4,5]. Food and beverages are repeatedly introduced to the oral cavity causing changes in the color of natural teeth as well as dental restorations [6]. Moreover, smoking cigarettes has been considered among the most harmful stimuli to oral health in general [7,8] and to the color of teeth and restorative materials in particular [9,10,11,12,13]. Tobacco smoking has often been accounted for causing dental staining [11]. Dalrymple et al. stated that exposure to cigarettes caused a large change in the color of enamel and dentine, demonstrated as increased staining compared with a non-exposure control [10,12]. In contrast, there was evidence showing that cigarette smoke caused a little bit more discoloration on ceramic than the control while studying the impact of cigarette smoke on dental ceramics [13].

According to the World Health Organization, cigarettes are the most widely used form of tobacco [7]. Recently introduced products such as electronic cigarette devices (ECDs) have gained popularity in the past few years. These electronic nicotine delivery systems (ENDS) are devices that create an aerosol by heating a liquid containing propylene glycol and flavoring agents [6,14]. The produced aerosol is further inhaled by the user. Although e-cigarettes do not contain tobacco, they still remain detrimental to health, and the long-term impacts of using them or being exposed to them remain a concern [7,14]. Manufacturers often advertise these novel products as having no smell or causing yellow teeth [15]. In fact, prior in-vitro research discovered that exposure to e-cigarettes altered enamel color more than the non-exposure control group [10,12]. In another study exploring the relationship between various flavors of e-cigarettes and their consequent effect on discoloration, the authors demonstrated that all tested flavors resulted in staining of enamel [16]. E-cigarettes have also negatively affected the color and the translucency of composite resin restorative materials, making them appear darker [17]. However, there is some suggestion that e-cigarette aerosol exposure has some influence on the staining of dental ceramics, with little evidence of dose-dependent change linking nicotine concentration to the degree of color change.

The rapid increase in the consumption of electronic cigarettes worldwide necessitates studying the impact of such stimuli on the color stability of the wide variety of dental ceramic materials available nowadays. Therefore, in this in vitro analysis, the effect of electronic cigarette devices/Vape (ECDs) on modern-day esthetic dental ceramics was investigated by recording the CIELAB coordinates (L*a*b*) and calculation of total color difference (ΔE) values using a spectrophotometer.

## 2. Materials and Methods

The study was carried out at King Saud University’s College of Dentistry’s Department of Prosthodontics and CDRC in Riyadh, Saudi Arabia. Before the study began, the King Saud University, College of Dentistry Research Center’s ethical committee granted its approval (Registration # FR0638).

### 2.1. Selected Materials

Based on market availability and ease of preparation, five regularly used restorative dental ceramic materials (Pressable ceramics (PEmax); Pressed and layered ceramics (LEmax); Layered zirconia (LZr); Monolithic zirconia (MZr); and Porcelain fused to metal (PFM)) of A1 shade were selected. Table 1 lists the specifics of the materials.

### 2.2. Sample Size Calculation

The specimens were prepared in accordance with the sample size determined as follows for each of the five test group materials. There were 15 (*n* = 15) samples in each group, for a total of 75 (N = 75) samples across all test groups (Table 1). The G-power software (G* Power 3.1.9.7, Dusseldorf, Germany) was used for the computation of the total sample size with an effect size of 0.45, a power of 0.85, and the level of significance set at 0.05 or less.

### 2.3. Sample Fabrication

**Figure 1:**
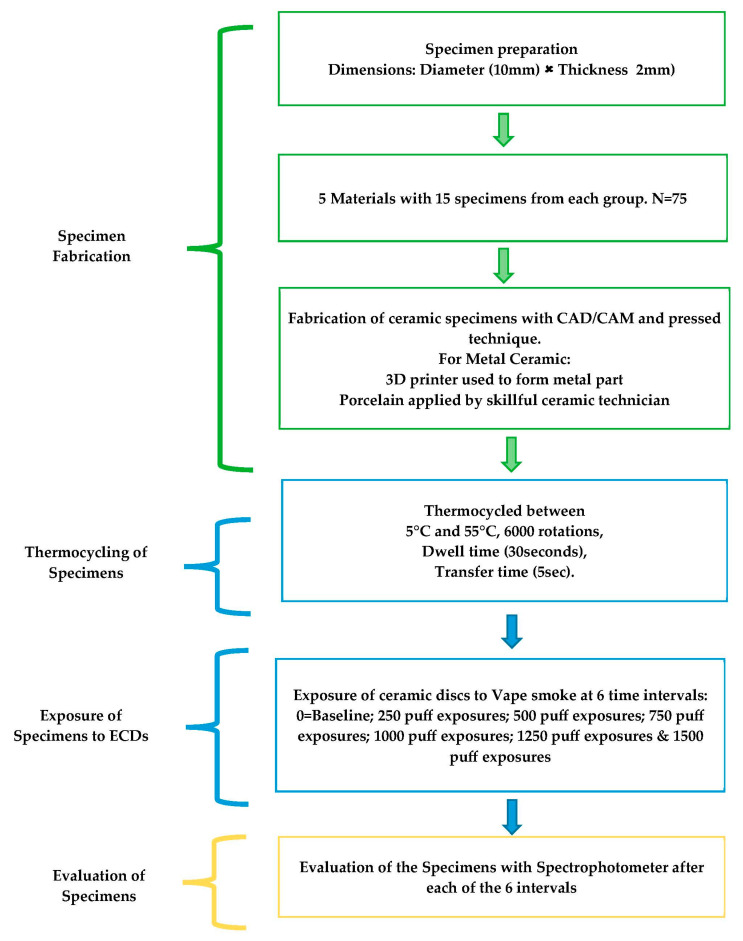
Methodology protocol followed during the study.

The test group samples were made up of standard-sized discs with a 2 mm thickness and 10 mm diameter. Each material group’s samples were manufactured in accordance with the manufacturer’s instructions. With the aid of an electronic caliper, the thickness was standardized. The process of making the discs required building the specimens using software, then machining the specimens for the different material groups using CAD/CAM technology. Professional technicians added the porcelain powder to the framework materials for the specimens made with layering ceramics, and two experts then evaluated each manufactured ceramic disc (Figure 1).

### 2.4. Thermocycling of Specimens

For 24 h, the manufactured specimens were kept in distilled water at 37 °C to replicate a clinical setting or an oral cavity condition. Following that, samples were aged by being stored in a machine for thermocycling (Huber, SD-Mechatronik-Thermocycler, Westerham, Germany) for 6000 rotations with a dwell-time of 30 s and a transfer-time of 5 s at temperatures between 5 °C and 55 °C.

### 2.5. ECDs/Vape Puff Exposures

**Figure 2:**
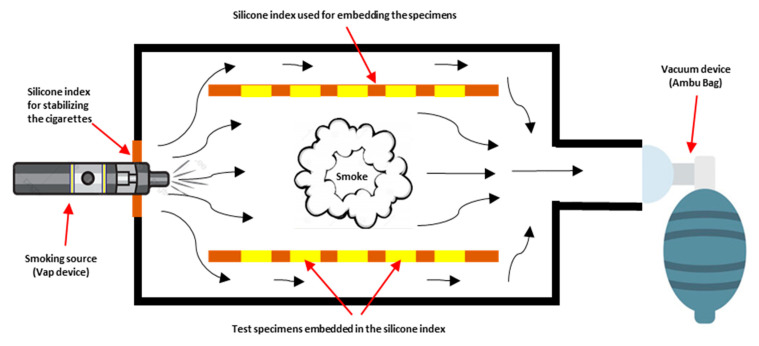
Schematic representation of the methodology used for introduction and exposing the vape smoke to the tested ceramic discs.

**Figure 3:**
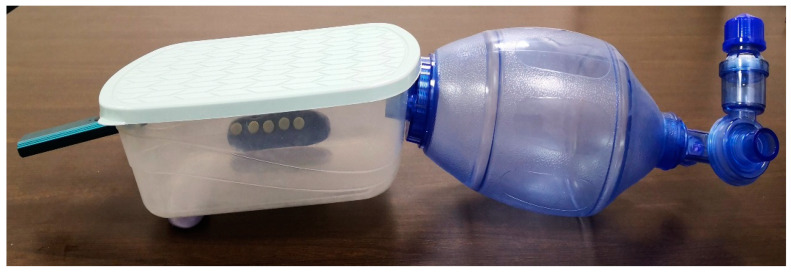
Customized chamber device used for vape smoke exposure.

An artificial lung vacuum system was used to guide smoke into a specially designed plastic chamber as part of a custom chamber equipment (Figure 2 and Figure 3). A polyvinyl-siloxane putty index (ExpressTM XT Putty Soft VPS, 3MTM, 3M, Riyadh, Saudi Arabia) that was positioned within the box at a distance of 10 cm from the vape smoke stabilized the specimens. In order to simulate the intraoral scenario of ceramics being exposed to smoking, the specimens were positioned vertically on both sides of customized chamber. The specimens were purposefully not placed horizontally to prevent the unrealistic adhesion of vape juice dust on the specimens.

ECDs/Vape device (Caliburn-X POD System, Shenzhen Uwell Technology Co., Ltd., Shenzhen, Guangdong Province, China) and refill juice (VGOD, LUSH-ICE, XL-Vape, SaltnicLabs Line, Torrance, CA, USA) were used for the puff generation in the study. The device, juice, and concentration of juice were selected based on their high consumption among the consumers according to the survey of the different devices and juice flavors available on the market. The vape device was attached to one end of the customized chamber, and the Ambu bag (PVC Manual Resuscitator Ambu Bag, Adult Size, Model NO.TW8311, Xiamen Winner Medical Co., Ltd., Xiamen, Fujian, China) was attached at the other end of the chamber. The electronic device was turned on before the start of each exposure. The Ambu bag was pressed completely and then released, which initiated the vape device to introduce the smoke in the chamber from all around. The e-cigarette batteries were kept at room temperature after being fully charged. The aerosol was removed from the atomizer by the vacuum flow, which then made it possible for the aerosol to be trapped in the chamber. The ceramic discs were exposed to vape smoke till the smoke was present (approximately for 40 to 60 s). This procedure was repeated until the targeted exposure puffs were achieved for each of the six exposure cycles as follows: 0 = Baseline; 250 puff exposures; 500 puff exposures; 750 puff exposures; 1000 puff exposures; 1250 puff exposures; and 1500 puff exposures.

### 2.6. Spectrophotometric Analysis

**Figure 4:**
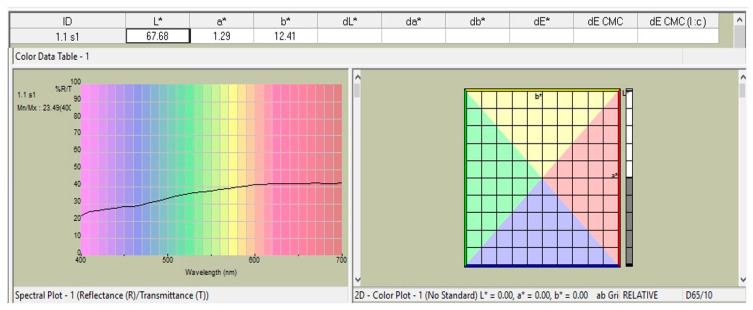
A spectral diagram that displays the “L*a*b*” values of one of the five test ceramic specimens obtained from a spectrophotometer.

Each sample’s color was measured using a spectrophotometer (LabScan-XE^®^, Hunter-Lab, Sunset Hills Road, Reston, VA, USA), which was also used to calculate “L*a*b values”. ‘L*’ stands for lightness, ‘a*’ for the axes of green (−a), red (+a), and blue (−b), and ‘b*’ for the axes of yellow (+b). The spectrophotometer sensor was placed over each prepared sample while it was against a black background, and three observations—or three readings—were made for each sample (Figure 4).

By using the formula below to determine the overall color differences (ΔE) from “L*a*b*” values obtained with a black background, the stability of color was assessed;

‘[ΔE = [(ΔL*)2 + (Δa*)2 + (Δb*)2]^1/2^]’

where the

ΔL = L (Different time exposures) − L (Baseline reading)

Δa = a (Different time exposures) − a (Baseline reading)

Δb = b (Different time exposures) − b (Baseline reading)

### 2.7. Statistical Tests

Statistical software for social sciences was used to compile and analyze all of the data (SPSS; v23; SPSS Inc., Chicago, IL, USA). The mean values of the L*a*b* color coordinates and the derivation of ΔE from the L*a*b* values using the aforementioned formula were the descriptive variables for each specimen of the ceramic disks. The statistical study compared ΔE mean values with 95% confidence intervals for all specimens using one-way analysis of variance (ANOVA) and Tukey’s posthoc testing for multiple comparisons. The significance cutoff was established at α < 0.05.

## 3. Results

Table 2 provides descriptive data (mean and standard deviation of “L*” values obtained with the spectrophotometer) for the five tested materials (N = 75). The MZr had the lowest value (57.46), and the PEmax had the highest value (69.28) before the specimens were exposed to ECDs. Despite the fact that all of the materials were constructed using shade A1, there were significant differences in the lightness of the materials tested at baseline before exposure. With the exception of the PEmax, which showed no change in its lightness value (*p* = 0.11), all of the tested materials’ lightness considerably changed following exposure to 250 puffs. When the number of puffs rose, the lightness value continued to change. The fluctuations in the L* value, however, were inconsistent. The layered ceramics LEmax and LZr showed the greatest change in lightness L* from their baseline measurements after 1500 puffs (59.61 to 64.53 and 61.04 to 72.95, respectively) (Table 2).

Table 3 displays changes in the a* value following exposure to ECDs, with the most notable shift occurring after 500 puffs, with changes leaning more toward the green zone of the red-green axis. With the exception of the PFM group, all six levels of values for “a*” for all the groups at the six levels of puff exposures were interestingly negative. After exposure to 1000 puffs, pushing it to the red zone of the red-green axis, the largest a* value decrease (−2.59) and rise (1.32) were seen in the MZr and PFM, respectively. All of the studied materials turned greener than the baseline at the conclusion of the exposure cycles, or after exposure to 1500 puffs, with the exception of the PFM group. The LZr group had the largest decline after 1500 puffs when compared to the baseline, with a decrease of 0.25 units in the direction of the axis’ green zone.

In all groups, with the exception of the PEmax group (*p* = 0.366), Table 4 demonstrates significant changes (*p* < 0.05) in the b* value following exposure to ECDs at the six different puff exposure intervals. After exposure to 250 puffs, the LEmax, LZr, and MZr groups significantly shifted toward the blue zone of the yellow–blue axis, whereas the PFM group shifted toward the yellow zone. It was interesting to observe that, with the exception of the MZr group, which had values at various levels that were in negative readings, all of the groups’ readings of “b*” at the six puff exposure levels were positive readings. The biggest b* value change was recorded in the PFM (13.33) after exposure to 1000 puffs, moving it to the yellow zone of the yellow–blue axis, while the highest b* value drop was seen in the MZr (−0.80) after 750 puffs. All tested materials turned yellower than the baseline at the end of the exposure cycles or after exposure to 1500 puffs.

Descriptive data (mean and standard deviation) are provided in Table 5 for the “ΔE” values derived from the “L*a*b*” values at various exposure intervals in relation to baseline readings for the five materials that were evaluated (N = 75). All test materials showed significant color variations (ΔE) upon exposure to ECDs (*p* < 0.05). The LZr group displayed substantially high ΔE values at each of the different puff exposure intervals, with the highest ΔE value (13.67) occurring after 1500 puffs. At 1500 puffs, the LEmax group likewise showed an elevated ΔE value (4.69). Despite being statistically significant, the ΔE values in the PFM group were rather low at 250 and 500 puffs (0.85 and 0.97, respectively) before steadily rising to 1.92 at 1500 puffs. All test groups displayed their greatest ΔE values at the conclusion of the exposure cycles.

## 4. Discussion

By recording the “CIE-L*a*b*” color coordinates, which are regarded as the complete numerical legend of the shade and obtained by utilising a spectrophotometer, and calculating the total color difference (ΔE) values [18], the present research evaluated and compared the effects of ECDs/Vape on the color changes of contemporary esthetic dental ceramics. In the investigation, a novel way of subjecting the specimens to vaping in a specially created closed chamber was used. The values were recorded for various Vape puff exposures at six different time intervals. The study’s findings disproved the null-hypothesis that there was no change in the color values after exposure to ECDs by showing substantial alterations in the recorded L*a*b* and ΔE values of the ceramic materials tested after exposure to the ECDs at various test time intervals.

The methodology adopted in the current study is distinctive and has been applied in other research projects of a similar nature [17,18]. In order to imitate the sucking pressure of the lungs and the entry of aerosols comparable to the oral cavity, the use of the Ambu bag for the formation of sucking pressure instead of a vacuum machine for the introduction of vaping aerosols to the chamber was an addition. To replicate the presence of aerosols in the oral cavity, aerosols were added, and the specimens were exposed to them from all directions. A legitimate and well-documented methodology for conducting color research on dental materials uses a spectrophotometer to record the CIE-L*a*b* color coordinates [19]. The readout of this instrument is unaffected by ambient light because it measures the amount of light reflected from the samples and collected throughout a complete spectrum of wavelengths. The high level of agreement seen in its use serves as confirmation of the validity of this methodology for in vitro research investigations on color and hues in dental sciences. These devices are rarely used to measure the color of teeth in dental offices since they are difficult, expensive, and knowledge-intensive [17,18,19].

Vaping is harmful to the teeth, gums, and associated structures [14]. Bacterial growth in the mouth is caused by e-cigarette vapor exposure on a regular basis. Cavities, periodontal disease, and tooth decay are all related to this. Inflammation of the gums, dry mouth, halitosis, and numerous other oral problems are also brought on by it [20]. It is obvious that vaping could have a harmful impact on periodontal health and general health and hasten the onset of lung disorders [21]. ECDs use an “atomizer”, a device that heats an e-liquid. Water, flavorings, propylene glycol, and glycerin make up the e-liquid, which also comes in a range of nicotine levels and nicotine-free choices. The lack of combustion means that “vaping” emits less harmful compounds than traditional smoking since when a device is engaged, vapors condense into an aerosol that the user inhales [22]. ECDs may offer a less hazardous supply of nicotine than regular cigarettes, according to some research, but there is still little proof to back this up over the long run. Users of ECDs are particularly vulnerable to dangerous metals like tin, silver, aluminum, chromium, nickel, and mercury that are produced when the e-liquid oxidizes [20,21,22]. Despite the lack of a systematic evaluation technique, studies have shown significant diversity in ECDs products, yielding contradictory and inconsistent results. Although there are no federal regulations governing their creation, sale, or usage in the United States [23], some countries, including Brazil, Singapore, Belgium, Uruguay, and others, have banned the sale of ECDs due to their unproven safety.

Traditional cigarette smoking is known to discolor teeth and change the color sta-bility of many dental materials, making people unhappy with how they look [9,10,11,12,13,24]. Few studies that give data on the impact of the aerosols produced by ECDs on hard dental structures such as enamel have been published in the literature [16,25]. The results of those studies showed that the ECDs do produce changes in the color of the teeth [16,25]. However, there is limited published information about how the aerosols generated by ECDs devices damage contemporary dental ceramics in terms of their color changes [13]. Theoretically, extrinsic stains such as ECDs aerosols should not affect the color of current dental ceramics and should respond similarly, irrespective of their composition [17]. In contrast, the results of this study demonstrated some fluctuations in the L*a*b* and ΔE values, resulting in some changes in the hue of the ceramics between different time intervals. This was in agreement with an earlier study using electronic nicotine delivery systems (ENDS), where significant color changes were observed after exposing the ceramic group (IPS Empress) to nicotine aerosols [13]. However, the earlier study was conducted at one time interval (equivalent to 700 puffs) using one type of ceramic material, unlike the present investigation, which measured color variations at six different time intervals using five ceramic materials; therefore, the comparison on the basis of exposure duration is not possible at this point. Most of the color values measured in the current study at the six time intervals revealed that the color of the ceramics changed at variable degrees depending on the ceramic material used as well as the time interval.

Although the present study’s findings demonstrated a color change in response to exposure to ECDs on the tested ceramics, such changes seem to be less than those caused by traditional smoking, as reported in previous studies [11]. The research we have indicates that cigarette smoke has a major impact on color stability. In comparison, though, there is little evidence that electronic cigarettes have less color change which can be quickly reversed under clinically acceptable criteria [26]. In a study by Dalrymple et al. 2018, the effect of conventional as well as ECDs on the color of enamel was compared, and their results revealed that conventional cigarettes significantly increased the level of bovine enamel sample discoloration, whereas exposure to the ECDs resulted in lower values compared to the controls [10]. This might be because combustion by-products from burning the chemicals in traditional cigarettes have a tendency to stick to surfaces [26]. Similarly, Schelkopf et al. have noted large amounts of cigarette smoke residue on the surfaces of the ceramic samples exposed to conventional cigarettes in their investigation and have linked the high reported ΔE values to the existence of such residues [27]. The total color difference (ΔE), is proven to be a gold standard of measurements used in color science for evaluating and contrasting color changes. ΔE < 1 cannot be seen with the unaided eye, ΔE < 3.3 can only be seen by an experienced dentist, and ΔE > 3.3 may be seen by anybody and is clinically undesirable, according to color changes in aesthetic restorations [28]. In the current investigation, the ΔE levels did change, and the most significant modifications of ΔE > 3.3 were observed after 1500 puff exposures for three of the investigated materials, namely, LEmax, LZr, and MZr. The ΔE for the PEmax and PFM was less than 3.3. The variation in ΔE values between the different materials in response to ECDs is probably related to material composition and structure, suggesting that color stability is material dependent. Dental glass ceramics are materials that consist of a glass matrix and crystalline phase. The relationship between the crystal size and their distribution with the glassy matrix crystals influences the color and optical of these ceramic materials [5,29]. The LZr (Zolid LT) zirconia core used in the current study is classified as 3Y-TZP, which is composed of 3 mol% yttria partially stabilized tetragonal zirconia polycrystal with >15% cubic-phase zirconia, while the MZr (Zolid gen-x) is an M4Y-PSZ with 4 mol% yttria partially stabilized zirconia having >50% cubic-phase zirconia [30,31]. This alteration in crystal structure reduced the residual pores and impurities, which, when present, produced volumes of different refractive indices and induced light scattering on the surface, influencing the final color and resulting in inferior optical properties [32]. The addition of cubic-phase zirconia enhances light transmission, according to Zhang and Lawn [5,31]. On the other hand, the Emax (IPS press) ceramic is composed of a dense and uniform distribution of lithium disilicate crystals with a 70% crystal volume (Li_2_SiO_3_) embedded in a glassy matrix and having crystal diameters between 0.2 and 1 m [5,31]. Generally, enhanced color characteristics and higher translucency are observed with a smaller crystalline ratio [33], which could explain the favorable performance of the PEmax material in the present study in terms of ΔE < 3.3. Notably, the PFM group had the fewest changes in ΔE values, making it the material least influenced by ECDs. The existence of a metal core, which prevents the effect of aerosols from one side of the specimens, could be one reason for the least alterations in the PFM group, as opposed to the other more translucent materials, which have the effect of aerosols on both sides of the specimens.

It is important to emphasize and talk about a few of the methodological techniques used in this investigation. The use of ECDs differed from traditional cigarette smoking in several ways, including the number of puffs taken, the length of each puff, the time between puffs, and the amount of juice or beverage swallowed [34]. According to certain research, an average person with ECDs takes somewhere between 25 and 140 puffs each day [35]. The 250 puffs total per week were taken into consideration for this investigation and used for each test period. The time it took to inhale a puff was determined by the Ambu bag’s one-time suction (about 3–4 s), and the subsequent puff was started once the smoke from the previous one had settled, which took between 40 and 60 s. ECDs devices come in a wide range of battery types, nicotine delivery systems, and liquid heating temperatures, all of which may affect particle distribution even when following a set puffing routine [36]. The physicochemical characteristics of the aerosols and their metal contents are altered by the e-cigarette’s heating settings, potentially harming the oral and respiratory systems of users. The aerosol produced at high power and temperature, in particular, has a more yellowish hue and a higher viscosity, which enables it to attach to the tooth surface for a longer period of time and perhaps speed up discoloration [37]. As a result, outcomes might only be applicable to particular devices. This makes it challenging to standardize a procedure for laboratory studies and additional result comparisons.

Due to various restrictions imposed by the in vitro design, care must be used when interpreting the current findings. Although every effort was taken to assure the uniform preparation of the specimens, it is impossible to completely rule out the possibility of human error. Additionally, it did not analyze different nicotine juice brands, strengths, and flavors, nor did it simulate brushing against specimens while they were exposed to smoke. Additionally, the absence of the oral environment, including the absence of hard (teeth) and soft tissues (lips and tongue) around, as well as the saliva as a medium, may become the cause of some errors. It is critical to continue the research in this area, because of lack of standardizing the testing procedures to assess the use of ECDSs and its implications in clinical dentistry. Setting up an experimental design to study the effect of aerosols or smoke on dental restorative material is quite challenging. Nonetheless, this study attempted to evaluate color changes in novel dental ceramics exposed to ECDs aerosols in an in vitro setting, and despite certain limitations, this study offered methodological data that can be a useful foundation for future research studies in this field to be more uniformly conducted.

Future comparative research should therefore take into account standardization of puff duration, refilling frequency for the e-liquid, and inter-puff interval time. The authors also suggest employing the CIEDE2000 (E00) formula in future studies for investigating color differences, as there are reports in the literature about its superiority in terms of human perception of minor color variations [38]. Last but not least, this study did not replicate several additional variables that can affect color change, such as dental brushing, eating practices, and concurrent smoking habits. Additionally, the authors would like to recommend comparing the effect of conventional smoking versus ECDs smoking on the color of the novel dental ceramics.

## 5. Conclusions

Within the bounds of the study’s limitations and in light of the findings, it can be said that vaping or electronic cigarette devices (ECDs) can noticeably alter the color of the dental ceramics used in the study. Clinically, the effect of smoke exposure from ECDs on the color of the examined specimens for the time period that the specimens were exposed resulted in modest to moderate modifications on the color of dental ceramics, affecting the esthetics. In comparison to the other dental ceramics evaluated, Pressable Lithium Disilicate Ceramics (PEmax) demonstrated improved color stability.

## Figures and Tables

**Table 1:** Dental ceramics tested in the present research investigation.

S. No.	Group	Material	Trade Name	Manufacturer	Lot Number
1.	PEmax	Pressable ceramic	IPS e.max Press	Ivoclar Vivadent AG Schaan, Principality of Liechtenstein.	A1 = Y05769 B1 = Y44299; C1 = 605274
2.	LEmax	Pressable	IPS e.max Press	Ivoclar Vivadent AG Schaan, Principality of Liechtenstein.	A1 = Y05769 B1 = Y44299; C1 = 605274
		Ceramic layer	IPS InLine Dentin	Ivoclar Vivadent AG Schaan, Principality of Liechtenstein.	A1 = W39158; B1 = W40241; C1 = R71757
3.	LZr	Zirconia core	ZI, LT, Zolid * CAD/CAM material	Amann Girrbach, Koblach, Austria.	1905001
		Layering ceramic	IPS Inline Dentin	Ivoclar Vivadent AG Schaan, Principality of Liechtenstein	A1 = W39158; B1 = W40241; C1 = R71757
4.	MZr	Zirconia block	Zolid gen-x * CAD/CAM material	Amann Girrbach, Koblach, Austria.	1905001
		Frame shade	Dying liquid	3M ESPE, MN 55144 USA	A1 = 68346 B1 = 68574 C1 = 68579
5.	PFM	Metal	Starbond easy powder 30	Scheftner dental alloys, S&S Scheftner GmbH, Germany	0223060919
		Ceramic	IPS InLine Dentin	Ivoclar Vivadent AG Schaan, Principality of Liechtenstein.	A1 = W39158; B1 = W40241; C1 = R71757

* Computer aided design and computer aided manufacturing.

**Table 2:** The five test materials’ mean and standard deviation of “L” values at different “Vap” time exposures.

Exposure Timing	PEmax		LEmax		LZr		MZr		PFM	
	Mean	SD *	Mean	SD	Mean	SD	Mean	SD	Mean	SD
0 **	69.28	4.41	59.61	3.31	61.04	1.43	57.46	1.09	66.84	1.46
250	70.55	1.74	61.85	0.96	71.112	0.57	59.07	0.82	67.26	1.20
500	70.86	1.20	61.9	1.08	71.29	0.43	59.25	0.60	67.60	1.08
750	71.02	1.07	62.03	0.70	71.97	0.65	59.66	0.45	67.68	1.11
1000	71.11	0.87	62.85	0.57	72.34	0.37	60.33	0.47	68.12	1.58
1250	71.14	1.41	63.48	0.70	72.73	0.52	60.37	0.32	68.43	1.02
1500	71.39	1.11	64.53	2.24	72.95	0.82	61.01	0.47	68.61	1.38

* SD = Standard deviation; ** 0 = Before exposure.

**Table 3:** The five test materials’ mean and standard deviation of “a” values at different “Vap” time exposures.

Exposure Timing	PEmax		LEmax		LZr		MZr		PFM	
	Mean	SD *	Mean	SD	Mean	SD	Mean	SD	Mean	SD
0 **	−1.00	0.13	−2.01	0.14	−0.54	0.09	−2.28	0.26	1.12	0.23
250	−1.02	0.11	−2.04	0.09	−0.54	0.21	−2.39	0.26	1.17	0.21
500	−1.04	0.08	−2.21	0.04	−0.67	0.22	−2.49	0.27	1.16	0.23
750	−1.11	0.07	−2.20	0.03	−0.62	0.22	−2.59	0.28	1.12	0.22
1000	−1.00	0.10	−2.20	0.03	−0.67	0.22	−2.44	0.24	1.32	0.22
1250	−1.06	0.07	−2.11	0.04	−0.64	0.21	−2.41	0.24	1.17	0.23
1500	−1.11	0.09	−1.71	0.31	−0.81	0.23	−2.39	0.27	1.16	0.23

* SD = Standard deviation; ** 0 = Before exposure.

**Table 4:** The five test materials’ mean and standard deviation of “b” values at different “Vap” time exposures.

Exposure Timing	PEmax		LEmax		LZr		MZr		PFM	
	Mean	SD *	Mean	SD	Mean	SD	Mean	SD	Mean	SD
0 **	8.59	0.42	4.49	0.37	10.17	0.36	−0.36	0.68	12.62	0.42
250	8.63	0.58	4.19	0.36	9.80	0.45	−0.52	0.71	12.76	0.45
500	8.77	0.41	4.09	0.36	9.99	0.28	−0.49	0.80	12.74	0.44
750	8.62	0.35	4.05	0.40	10.08	0.28	−0.80	0.80	12.71	0.36
1000	8.92	0.30	4.28	0.32	10.13	0.30	−0.25	0.64	13.33	0.59
1250	8.74	0.35	4.82	0.35	10.30	0.26	−0.29	0.65	13.11	0.39
1500	8.71	0.44	5.89	0.251	10.72	0.30	0.18	0.70	13.04	0.43

* SD = Standard deviation; ** 0 = Before exposure.

**Table 5:** Calculated total color difference (ΔE) values for the five test materials at various ‘Vap’ time exposures.

Exposure Timing	ΔE					
	PEmax	LEmax	LZr	MZr	PFM	Anova *p* Value *
250	2.65	2.61	11.81	1.70	0.85	0.00
500	2.70	2.59	11.86	1.92	0.97	0.00
750	2.82	2.89	12.50	2.31	1.19	0.00
1000	2.83	3.41	12.83	2.89	1.75	0.00
1250	3.05	3.92	13.26	2.93	1.72	0.00
1500	3.09	4.69	13.67	3.64	1.92	0.00
Anova *p* value **	0.999	0.000	0.002	0.001	0.001	

* For comparisons between the groups at each exposure time, *p* < 0.05 was considered significant. ** The *p* value for comparisons within each group at various exposure times was significant at *p* < 0.05.

## Data Availability

Data are accessible from the corresponding author upon request.

## References

[1] Blatz M.B., Chiche G., Bahat O., Roblee R., Coachman C., Heymann H.O. (2019). Evolution of Aesthetic Dentistry. J. Dent. Res..

[2] de Matos J.D.M., Lopes G.R.S., Queiroz D.A., Nakano L.J.N., Ribeiro N.C.R., Barbosa A.B., Anami L.C., Bottino M.A. (2022). Dental Ceramics: Fabrication Methods and Aesthetic Characterization. Coatings.

[3] Warreth A., Elkareimi Y. (2020). All-ceramic restorations: A review of the literature. Saudi Dent. J..

[4] Lamont R.J., Koo H., Hajishengallis G. (2018). The oral microbiota: Dynamic communities and host interactions. Nat. Rev. Microbiol..

[5] Alrabeah G., Shabib S., Almomen R., Alhedeithi N., Alotaibi S., Habib S.R. (2023). Effect of Home Bleaching on the Optical Properties and Surface Roughness of Novel Aesthetic Dental Ceramics. Coatings.

[6] Tanthanuch S., Kukiattrakoon B., Thongsroi T., Saesaw P., Pongpaiboon N., Saewong S. (2022). In vitro surface and color changes of tooth-colored restorative materials after sport and energy drink cyclic immersions. BMC Oral. Health.

[7] World Health Organisation Tobacco: Key Facts. https://www.who.int/news-room/fact-sheets/detail/tobacco.

[8] Zhang Y., He J., He B., Huang R., Li M. (2019). Effect of tobacco on periodontal disease and oral cancer. Tob. Induc. Dis..

[9] Zanetti F., Zhao X., Pan J., Peitsch M.C., Hoeng J., Ren Y. (2019). Effects of cigarette smoke and tobacco heating aerosol on color stability of dental enamel, dentin, and composite resin restorations. Quintessence Int..

[10] Dalrymple A., Badrock T.C., Terry A., Barber M., Hall P.J., Thorne D., Gaca M.D., Coburn S., Proctor C. (2018). Assessment of enamel discoloration in vitro following exposure to cigarette smoke and emissions from novel vapor and tobacco heating products. Am. J. Dent..

[11] Karanjkar R.R., Preshaw P.M., Ellis J.S., Holliday R. (2022). Effect of tobacco and nicotine in causing staining of dental hard tissues and dental materials: A systematic review and meta-analysis. Clin. Exper. Dent. Res..

[12] Dalrymple A., Bean E.J., Badrock T.C., Weidman R.A., Thissen J., Coburn S., Murphy J. (2021). Enamel staining with E-cigarettes, tobacco heating products and modern oral nicotine products compared with cigarettes and snus: An in vitro study. Am. J. Dent..

[13] Vohra F., Andejani A., Alamri O., Alshehri A., Al-Hamdan R.S., Almohareb T., Abduljabbar T. (2020). Influence of electronic nicotine delivery systems (ENDS) in comparison to conventional cigarette on color stability of dental restorative materials. Pakistan J. Med. Sci..

[14] Marques P., Piqueras L., Sanz M.J. (2021). An updated overview of e-cigarette impact on human health. Respir. Res..

[15] Vapex E-Cigarettes Vapex E-Cigarette Freestarter Kit. Truth in Advertising. https://www.truthinadvertising.org/vapex-e-cigarette-freestarter-kit/.

[16] Pintado-Palomino K., de Almeida C.V.V.B., Oliveira-Santos C., Pires-de-Souza F.P., Tirapelli C. (2019). The effect of electronic cigarettes on dental enamel color. J. Esthet. Rest. Dent..

[17] Alnasser H.A., Elhejazi A.A., Al-Abdulaziz A.A., Alajlan S.S., Habib S.R. (2021). Effect of Conventional and Electronic Cigarettes Smoking on the Color Stability and Translucency of Tooth Colored Restorative Materials: An In Vitro Analysis. Coatings.

[18] Habib S.R., Rashoud A.S., Safhi T.A., Almajed A.H., Alnafisah H.A., Bajunaid S.O., Alqahtani A.S., Alqahtani M. (2021). Variations in the shades of contemporary dental ceramics: An In Vitro analysis. Crystals.

[19] Paolone G., Pavan F., Guglielmi P.C., Scotti N., Cantatore G., Vichi A. (2022). In vitro procedures for color stability evaluation of dental resin-based composites exposed to smoke: A scoping review. Dent. Mater. J..

[20] Eaton D.L., Kwan L.Y., Stratton K., National Academies of Sciences, Engineering, and Medicine (2018). E-cigarette devices, uses, and exposures. Public Health Consequences of E-Cigarettes.

[21] Ebersole J., Samburova V., Son Y., Cappelli D., Demopoulos C., Capurro A., Pinto A., Chrzan B., Kingsley K., Howard K. (2020). Harmful chemicals emitted from electronic cigarettes and potential deleterious effects in the oral cavity. Tob. Induc. Dis..

[22] Papaefstathiou E., Stylianou M., Agapiou A. (2019). Main and side stream effects of electronic cigarettes. J. Environ. Manag..

[23] Kyriakos C.N., Filippidis F.T., Hitchman S., Girvalaki C., Tzavara C., Demjén T., Fernandez E., Mons U., Trofor A., Tountas Y. (2018). Characteristics and correlates of electronic cigarette product attributes and undesirable events during e-cigarette use in six countries of the EUREST-PLUS ITC Europe Surveys. Tob. Induc. Dis..

[24] Smew A.A.M., Yildirim G., Guven M.C. (2022). Effect of cigarette smoking on the color stability and surface roughness of two different denture base materials. Am. J. Dent..

[25] Haiduc A., Zanetti F., Zhao X., Schlage W.K., Scherer M., Pluym N., Schlenger P., Ivanov N.V., Majeed S., Hoeng J. (2020). Analysis of chemical deposits on tooth enamel exposed to total particulate matter from cigarette smoke and tobacco heating system 2.2 aerosol by novel GC-MS deconvolution procedures. J. Chromatogr. B.

[26] Paolone G., Pavan F., Mandurino M., Baldani S., Guglielmi P.C., Scotti N., Cantatore G., Vichi A. (2023). Color stability of resin-based composites exposed to smoke. A systematic review. J. Esthet. Restor. Dent..

[27] Schelkopf S., Dini C., Beline T., Wee A.G., Barão V.A.R., Sukotjo C., Yuan J.C.-C. (2022). The Effect of Smoking and Brushing on the Color Stability and Stainability of Different CAD/CAM Restorative Materials. Materials.

[28] What is Delta E? And Why Is It Important for Color Accuracy?. https://www.viewsonic.com/library/creative-work/what-is-delta-e-and-why-is-it-important-for-color-accuracy/.

[29] Comba A., Paolone G., Baldi A., Vichi A., Goracci C., Bertozzi G., Scotti N. (2022). Effects of substrate and cement shade on the translucency and color of cad/cam lithium- disilicate and zirconia ceramic materials. Polymers.

[30] Ban S. (2021). Classification and Properties of Dental Zirconia as Implant Fixtures and Superstructures. Materials.

[31] Zhang Y., Lawn B.R. (2018). Novel zirconia materials in dentistry. J. Dent. Res..

[32] Ziyad T.A., Abu-Naba’a L.A., Almohammed S.N. (2021). Optical properties of CAD-CAM monolithic systems compared: Three multi-layered zirconia and one lithium disilicate system. Heliyon.

[33] Zhao M., Sun Y., Zhang J., Zhang Y. (2018). Novel translucent and strong submicron alumina ceramics for dental restorations. J. Dent. Res..

[34] Primack B.A., Shensa A., Sidani J.E., Hoffman B.L., Soneji S., Sargent J.D., Hoffman R.M., Fine M.J. (2018). Initiation of Traditional Cigarette Smoking after Electronic Cigarette Use among Tobacco-Naïve US Young Adults. Am. J. Med..

[35] Wagener T.L., Avery J.A., Leavens E.L.S., Simmons W.K. (2021). Associated Changes in E-cigarette Puff Duration and Cigarettes Smoked per Day. Nicotine Tob. Res..

[36] Unger M., Unger D.W. (2018). E-cigarettes/electronic nicotine delivery systems: A word of caution on health and new product development. J. Thorac. Dis..

[37] Ko T.J., Kim S.A. (2022). Effect of Heating on Physicochemical Property of Aerosols during Vaping. Int. J. Environ. Res. Public Health.

[38] Gómez-Polo C., Montero J., Gómez-Polo M., Martin Casado A. (2020). Comparison of the CIELab and CIEDE 2000 Color Difference Formulas on Gingival Color Space. J. Prosthodont..

